# Looking back and looking forward

**DOI:** 10.1002/mgg3.374

**Published:** 2018-02-22

**Authors:** Maximilian Muenke, Suzanne Hart

**Affiliations:** ^1^ National Human Genome Research Institute National Institutes of Health Bethesda MD USA

Maximilian Muenke, Founding Editor‐in‐Chief

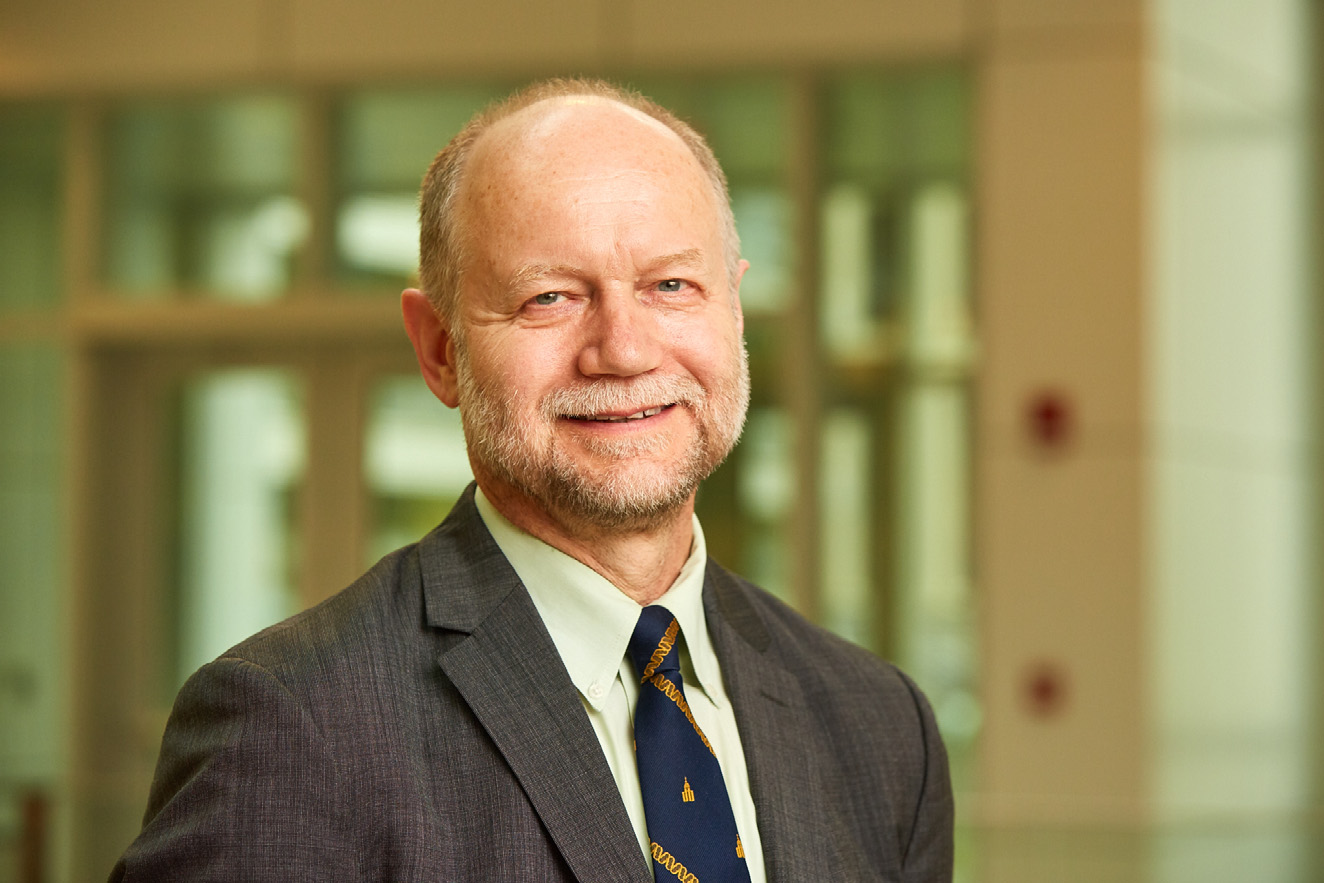

Suzanne Hart, Editor‐in‐Chief

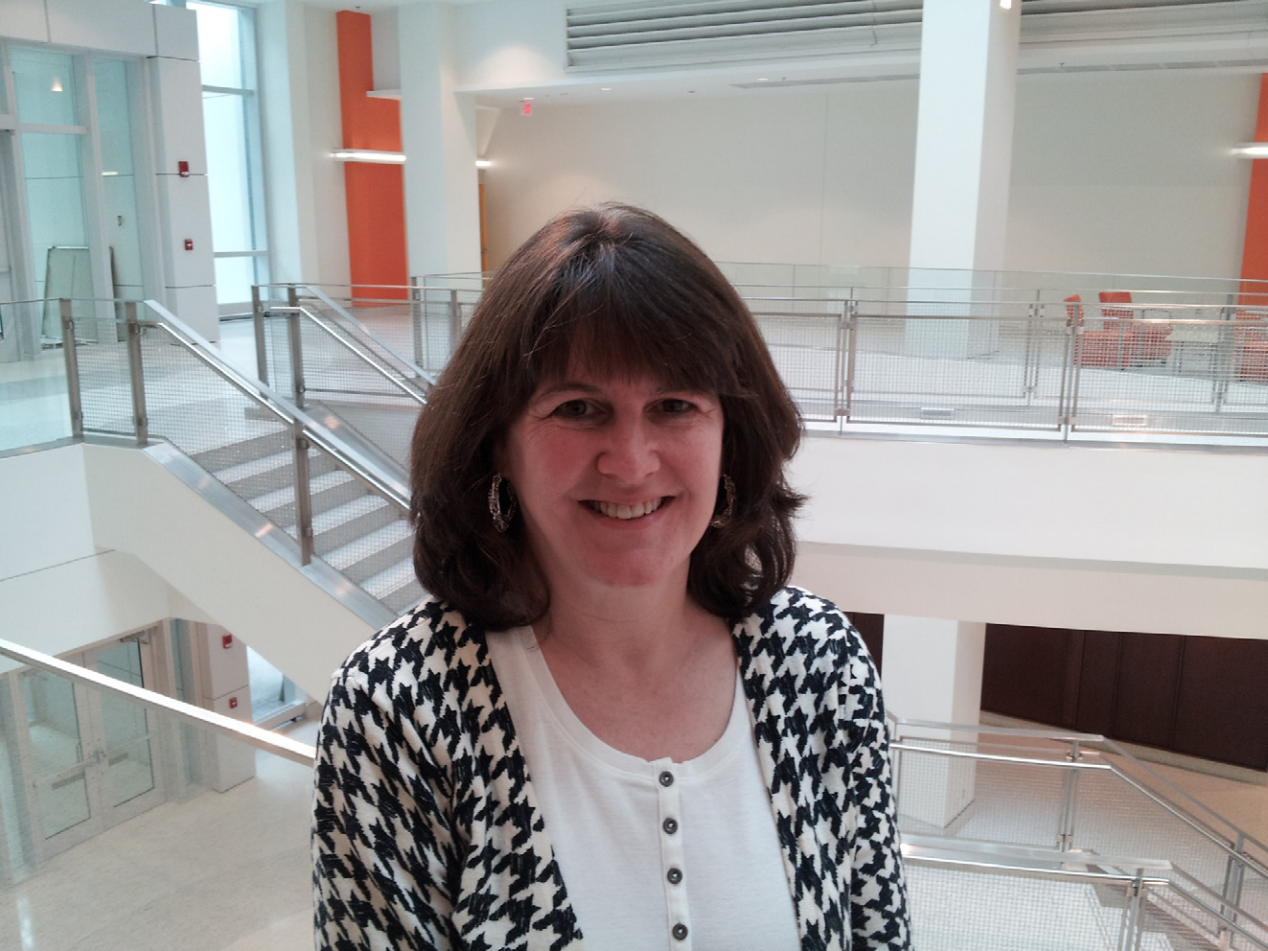

This year *Molecular Genetics & Genomic Medicine* (*MGGM*) turns 5 years old. For the editors of this journal it is time for some reflection, “looking back and looking forward”. The first issue appeared in May of 2013, 60 years after the publication by Watson and Crick's discovery of the double‐helical structure of DNA and 10 years after the completion of the Human Genome Project.

The work for this journal began 1 year earlier with the selection of the Founding Editor‐in‐Chief (EIC) Max Muenke, choice of a journal title, and the Aims and Scope of the journal. Initial options for titles included *Human & Medical Genetics*,* Medical Genetics & Genomic Medicine*, and a few others, before *Molecular Genetics & Genomic Medicine* became the top choice. Suzanne Hart was chosen as Deputy Editor‐in‐Chief in part because of her expertise that is different from the EIC. Twenty‐four colleagues (10 female and 14 male) with a wide range of expertise from 15 different countries were invited to serve on the *MGGM* Editorial Board.

The *Aims* and *Scope* of *MGGM* were and are broad, to encompass a wide range of topics including basic and translational science, technological advances, human and medical genetics, animal models of human disease, pharmacogenomics and translational medicine (Muenke, 2013). From the first Editorial to the Invited Commentaries in each issue, various aspects of genomic medicine have been illustrated: integration of genomic information into the delivery of health care (De Castro & Turner, 2017; Eng, 2013; Francke, 2013; Slavotinek, 2016; Solomon, 2016; Williams, 2015), educational opportunities (Hart & Hart, 2016; Korf, 2013; McCarthy, 2014; Muenke, 2016), genomic counseling (Middleton, Hall, & Patch, 2015; Ormond, 2013); diverse populations (Adeyemo & Rotimi, 2014), ethical considerations (Wilfond & Goddard, 2015; one of the 10 most highly cited articles in *MGGM*, see below), comparative genomics (Mullikin, 2014), and many more including specific groups of diseases: newborn screening (Levy, 2014); substance use disorders (Rutter & Volkow, 2014); hearing loss (Pandya, 2016); orofacial clefting (Adeyemo & Butali, 2017), and stuttering (Frierio‐Domingues & Drayna, 2017). Some of my personal (M.M.) highlights were those articles celebrating the sesquicentennial anniversary of Johann Gregor Mendel (De Castro, 2016; Opitz & Bianchi, 2015), including an essay by Mendel's great‐great‐grandnephew, the Augustinian monk Father Clemens Richter (Richter, 2015).

**Figure 1:**
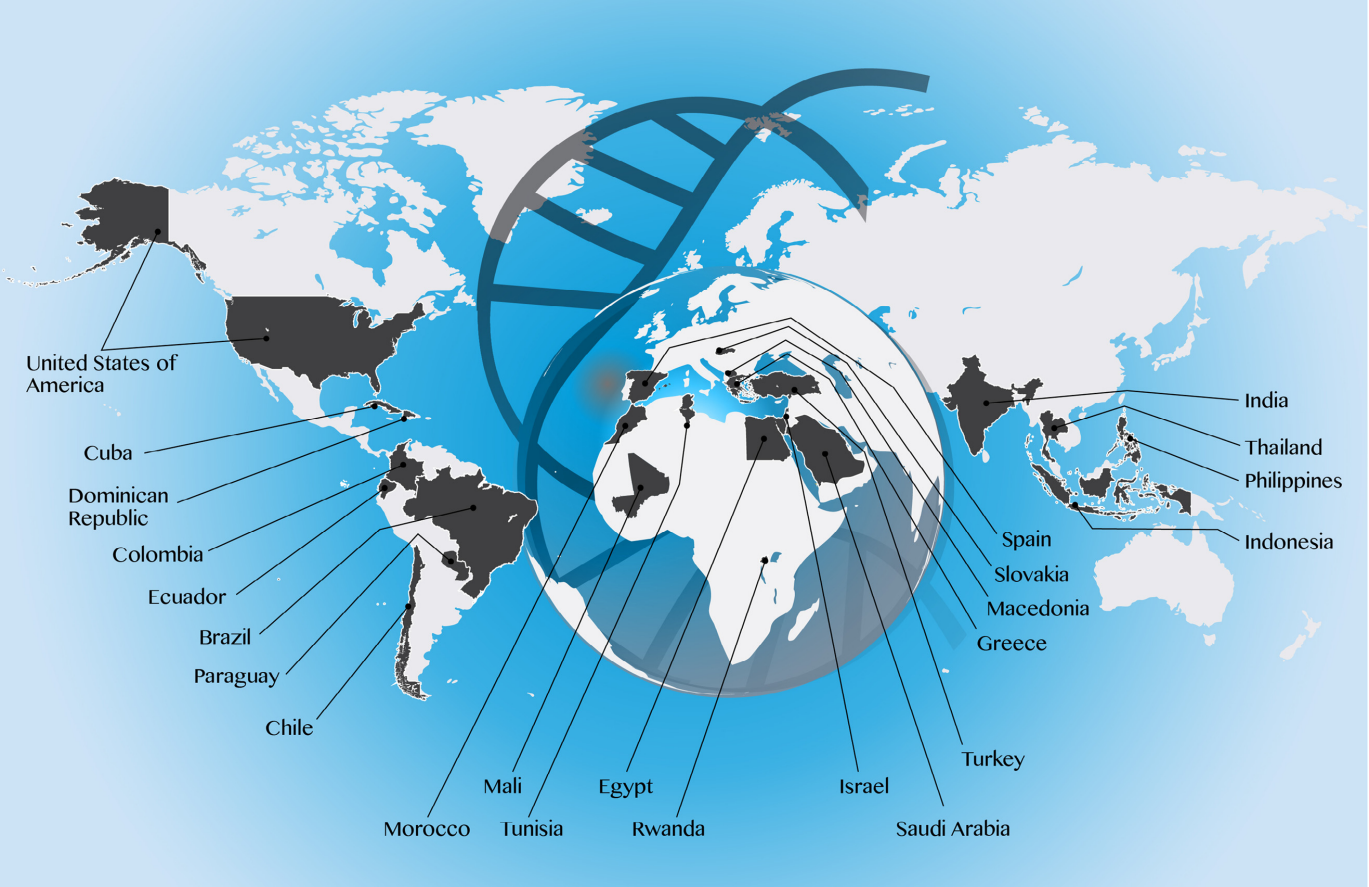
Diagram of countries and geographical localization on the world map for which a manuscript has been published or is in press

Another regular feature of *MGGM* that started in 2014, the series on “Genetics and Genomic Medicine Around the World”, is certainly among our favorites. It was our hope that this series “will be an invaluable sounding board for learning how different countries and cultures view and adopt genetic and genomic testing” (Hart & Muenke, 2014). To date, this has been the longest running such series when compared to any other genetics journal. We have learned from two dozen countries: small (Cuba, Ecuador, Macedonia, Paraguay, Rwanda, Slovakia) and large (India, Brazil, USA) and any size in between (Table 1; Figure 1). Working with colleagues around world on these articles has been enjoyable and one of the most rewarding aspects of being the editors for *MGGM*.

**Table 1:** Genetics and genomic medicine around the world

Africa	
Egypt	Temtamy and Hussen (2017)
Rwanda	Uwineza and Mutesa (2015)
Mali	Landouré et al. (2016)
Morocco	Belhassan, Ouldim, and Sefiani (2016)
Tunisia	Elloumi and Chaabouni (2018) (in press)
Middle East	
Saudi Arabia	Alkuraya (2014)
Israel	Zlotogora (2014)
Turkey	Özçelik (2017)
Europe	
Greece	Manoli and Fryssira (2015)
Spain	Pàmpols et al. (2016)
Slovakia	Kádaši and Cisárik (2015)
Macedonia	Sukarova‐Angelovska and Petlichkovski (2018) (current issue)
Asia	
India	Aggarwal and Phadke (2015)
Thailand	Shotelersuk, Limwongse, and Mahasirimongkol (2014)
Indonesia	Ariani, Soeharso, and Sjarif (2017)
Philippines	Padilla and Cutiongco‐de la Paz (2016)
Americas	
Cuba	Balbuena and Teruel (2017)
Dominican Republic	Estrada‐Veras, Cabrera‐Peña, and Pérez‐Estrella de Ferrán (2016)
Ecuador	Paz‐y‐Miño, Sacoto, and Leone (2016)
Colombia	De Castro and Restrepo (2015)
Brazil	Passos‐Bueno, Bertola, Horovitz, de Faria Ferraz, and Brito (2014)
Paraguay	Ferreira and de Herreros (2014)
Chile	Taucher (2015)
USA	Ferreira, Regier, Hadley, Hart, and Muenke (2017); Regier, Ferreira, Hart, Hadley, and Muenke (2017)

Last, but not least, are the unsolicited, peer‐reviewed articles. Many submissions come directly to *MGGM*, while others are referred from our many high‐profile supporter journals published by Wiley, such as *Human Mutation*,* Clinical Genetics*, and *American Journal of Medical Genetics* (*AJMG*). As submissions have grown since 2013, the number of accepted articles has also steadily increased. Dedicated peer review and editorial evaluation ensures that all published articles meet rigorous scientific standards. As was our hope when the journal started, the topics of the unsolicited, peer‐reviewed articles are broad, ranging from diagnosis to treatment as is well illustrated by the titles of the 10 most highly cited manuscripts (Table 2).

**Table 2:** Top 10 most highly cited articles in *MGGM* (as of January 10, 2018)

Times Cited	Authors and titles
26	Hirotsu, Nakagomi, Sakamoto, Amemiya, Mochizuki, et al. (2015): Detection of *BRCA1* and *BRCA2* germline mutations in Japanese population using next‐generation sequencing
20	Sofou et al. (2015): Whole exome sequencing reveals mutations in *NARS2* and *PARS2*, encoding the mitochondrial asparaginyl‐tRNA synthetase and prolyl‐tRNA synthetase, in patients with Alpers syndrome
19	Hirotsu, Nakagomi, Sakamoto, Amemiya, Oyama, et al. (2015): Multigene panel analysis identified germline mutations of DNA repair genes in breast and ovarian cancer
18	Yang et al. (2015): Taurodontism, variations in tooth number, and misshapened crowns in Wnt10a null mice and human kindreds
17	Sekulic et al. (2015): Personalized treatment of Sezary syndrome by targeting a novel CTLA4: CD28 fusion
11	De Kovel et al. (2016): Targeted sequencing of 351 candidate genes for epileptic encephalopathy in a large cohort of patients
11	Yigit et al. (2015): Mutations in *CDK5RAP2* cause Seckel syndrome
10	Kassab et al. (2016): *GATA5* mutation homozygosity linked to a double outlet right ventricle phenotype in a Lebanese patient
10	Wilfond and Goddard (2015): It's complicated: criteria for policy decisions for the clinical integration of genome‐scale sequencing for reproductive decision making
10	Ammar‐Khodja et al. (2015): Diversity of the causal genes in hearing impaired Algerian individuals identified by whole exome sequencing

All *MGGM* articles are deposited in PubMed Central^®^ (PMC) and are indexed and discoverable in PubMed/MEDLINE. *MGGM* is now in Web of Science (WoS) via indexing in Clarivate Analytics’ Science Citation Index Expanded (SCIE).

In January 2017, the Founding Editor‐in‐Chief of *MGGM* became the third Editor‐in‐Chief of the *American Journal of Medical Genetics Part A* (*AJMG*) (Muenke, 2017). Moving forward, Max Muenke stepped down as EIC and Suzanne Hart is the Editor‐in‐Chief of *MGGM* starting with the January 2018 issue.

What has happened in the field of molecular genetics and genomic medicine over the past 5 years? Highlighted below is just a small selection of topics covered by review articles in the areas critical to our field: (1) technological advances leading to progress in (2) pharmacogenomics, (3) precision medicine in general, and (4) cancer genomics specifically.

Technological advances enabling better and cheaper next generation DNA sequencing (Goodwin, Mcpherson, & Mccombie, 2016; Shendure et al., 2017), including single cell genome sequencing (Gawad, Koh, & Quake, 2016), have made the less‐than‐$1,000 price tag per genome a reality. Despite the availability of tens of thousands of reference genomes such as ExAC (http://exac.broadinstitute.org), ClinVar (http://www.ncbi.nlm.nih.gov/clinvar), and others (Tarailo‐Graovac et al. 2017), high throughput functional validation of variants of unknown significance will continue to be critical in order to determine mechanisms of disease (zebrafish: Hong et al., 2016; mouse: Li et al., 2015; Liu et al., 2017; fruit fly: Zhu, Fu, Nettleton, Richman, & Han, 2017). Lastly, advances in genome engineering such as the CRISPER/Cas9 systems and others have led to breakthroughs in applications to gene therapy and regenerative medicine (Katrekar, Hu, & Mali, 2017).

Guidelines for the use of pharmacogenomics in clinical practice are available through the Clinical Pharmacogenetics Implementation Consortium (CPIC) (Johnson & Weitzel, 2016; Verbelen, Weale, & Lewis, 2017). To date, inherited variations in at least 20 genes have been shown to affect more than 80 medications and are actionable in the clinic (Relling & Evans, 2015). Noninvasive prenatal testing (NIPT) allows for prenatal pharmacogenomics, a “promising area of research” in the near future (Dorfman, Cheng, Hebert, Thummel, & Burke, 2016).

“The essential job of precision medicine is to match the right drugs to the right patients” (Letai, 2017). Precision medicine and cancer genomics specifically, have had numerous successes “from preconception to adult medicine” (Ashley, 2016; Rehm, 2017) from personalized treatment of cystic fibrosis to chronic myeloid leukemia and many others. Including diverse populations in human genomics research has become a priority for funding agencies like the National Institutes of Health in the US or the Wellcome Trust in the UK (Hindorff et al., 2018). Geographical differences in disease susceptibility have been recognized (Sud, Kinnersley, & Houlston, 2017; Tan, Mok, & Rebbeck, 2016). For example, prostate cancer has a higher incidence and more aggressive course in men of African descent compared to those from Northern Europe. Similarly, differences in incidence of lung cancer, colorectal cancer, and breast cancer have been identified in patients from different countries. Looking forward, we expect that with a decrease in price for medical genome sequencing and easier identification of disease‐causing alleles, personalized pharmacogenomics and precision medicine will become a reality for many individuals over the next decade.

Looking back has been inspiring, but looking forward is exciting! If the past five years are any indication of the rapid pace of future research, then the next five years in the allied fields of molecular genetics and genomic medicine will be full of new discoveries that will facilitate an improved understanding of the genetic etiology of disease. Such discoveries could in turn provide tools for better patient understanding and disease prevention, or stimulate investment in applied research that might speed the delivery of targeted treatments to the patient bedside. It is within this exciting research environment that the journal *Molecular Genetics & Genomic Medicine* proudly finds its home. Our journal's Open Access publication policy fully supports the public sharing of scientific knowledge aimed at advancing these admirable goals. As the editors of *MGGM,* we are honored to be able to publish, and make publicly available, papers representing the widest possible range of human and medical genetics research, and to provide valuable commentaries and global insights on new research and treatment trends. We sincerely thank the Editorial Board, our esteemed peer reviewers, and most of all, the authors and readers of *MGGM* for this honor.

So, at the start of 2018, we are proud of the accomplishments of *MGGM* over the past five years and are looking forward to continuing to publish high quality articles in the field in both the *American Journal of Medical Genetics* and *Molecular Genetics & Genomic Medicine*. We wish our readers the very best in 2018.

Max and Suzanne
